# Structural and Biological Properties of Rhamnogalacturonan-I-Enriched Pectin Isolated from *Cardamine tangutorum* and *Cardamine macrophylla*

**DOI:** 10.3390/foods14132340

**Published:** 2025-07-01

**Authors:** Mei-Mei Qu Mo, Bo Li, Ding-Tao Wu, Jing Feng, Jing Wei, Yan Wan, Juan Li, Yuan Liu, Wen-Bing Li

**Affiliations:** 1 Qinghai-Tibetan Plateau Ethnic Medicinal Resources Protection and Utilization Key Laboratory of National Ethnic Affairs Commission of the People’s Republic of China, Sichuan Provincial Qiang-Yi Medicinal Resources Protection and Utilization Technology Engineering Laboratory, Southwest Minzu University, Chengdu 610225, China; 2 Key Laboratory of Coarse Cereal Processing of Ministry of Agriculture and Rural Affairs, Sichuan Engineering & Technology Research Center of Coarse Cereal Industralization, School of Food and Biological Engineering, Chengdu University, Chengdu 610106, Chinayanwan@cdu.edu.cn (Y.W.); 3 Institute for Advanced Study, Chengdu University, Chengdu 610106, China

**Keywords:** Shigecai, *Cardamine* spp., pectic polysaccharide, chemical structure, biological activity

## Abstract

*C. macrophylla* and *C. tangutorum*, collectively known as Shigecai in Chinese, are consumed as special and nutritious vegetables by the Tibetan, Qiang, and Yi communities in China. However, due to the insufficient knowledge of their phytochemical compositions and health benefits, the industrial utilization of these species in the food sector remains limited. Although Shigecai leaves contain substantial pectic polysaccharides, their chemical structures and biological activities remain unknown, which ultimately restricts their industrial utilization. Thus, to address this gap, this study systematically analyzed the chemical characteristics and biological functions of rhamnogalacturonan-I (RG-I)- enriched pectin from *C. tangutorum* (CTHDP) and *C. macrophylla* (CMHDP) leaves. The results demonstrate that Shigecai leaves are promising sources of RG-I-enriched pectin, with yields of 57.63–65.21 mg/g dry weight. In addition, both CTHDP and CMHDP exhibited highly similar chemical and structural properties, dominated by RG-I and homogalacturonan (HG) pectin regions, with RG-I ratios of 60.14–63.33 mol%. Furthermore, both samples demonstrated notable antioxidant ability, antiglycation activity, prebiotic potency, and immunoregulatory effects, which were strongly linked to their bound polyphenol content, uronic acid content, and molecular weight. These findings support the industrial utilization of Shigecai and establish Shigecai-derived RG-I-enriched pectin as a promising functional food ingredient.

## 1. Introduction

Pectin, a structurally diverse heteropolysaccharide, serves as a fundamental structural composition of plant cell walls, which is abundantly found in vegetables and fruits [1,2,3]. Its content varies significantly across plant tissues, accounting for approximately 35% of dicotyledonous cell walls, 2–10% of grasses, and 5% of woody tissues [3]. Structurally, pectin comprises three mainly distinctive domains within plant cell walls, including homogalacturonan (HG, about 60–65%), rhamnogalacturonan-I (RG-I, about 20–35%), and rhamnogalacturonan-II (RG-II, about 2–10%) [2,3,4]. HG typically comprises linearly polymerized α-1,4-linked D-galacturonic acid residues, where carboxyl groups may undergo C-6 methylation and/or O-2/O-3 acetylation. Based on the degree of esterification (DE), HG can be classified as high methyl-esterified (DE > 50%) pectin and low methyl-esterified (DE < 50%) pectin. RG-I features a repeating disaccharide backbone of galacturonic acid and rhamnose units, with the latter bearing neutral side chains (e.g., galactan, arabinan, or arabinogalactan). Similar to HG, RG-I’s galacturonic acid residues may also be acetylated at the O-2/O-3 positions [2,3,4]. Pectin exhibits excellent functional properties, such as gelation capacity, emulsification performance, and film-forming ability. These characteristics have led to its wide utilization in the food and agricultural industries as a gelling component, thickener, emulsifier, and composite film material [5,6]. Moreover, pectin demonstrates remarkable health-promoting properties, such as an intestinal prebiotic effect, immunomodulatory activity, anti-cancer effect, anti-inflammatory effect, the enhancement of immune barriers, the regulation of blood glucose and lipid metabolism, and wound healing ability [2,3,5,7,8]. These beneficial biological properties have enabled its potential applications in healthcare, particularly in drug delivery systems, tissue engineering biomaterials, healthcare products for regulating blood glucose, lipids, and cholesterol, and pectin-based pharmaceuticals [2,3,5]. Furthermore, the functional and health-promoting properties of pectin are largely determined by its structural characteristics, such as HG/RG-I ratio, molar mass, sidechain composition, degree of esterification, and uronic acid content [2,5,9]. Notably, RG-I-enriched pectin demonstrates enhanced biological activities, particularly in prebiotic function, immunomodulation, and anti-cancer effect [6,10]. These superior biological properties have driven growing research interest in the discovery of edible plant sources rich in the RG-I pectin domain [3,4,6].

*Cardamine tangutorum* O.E. Schulz and *Cardamine macrophylla* Willd., collectively known as Shigecai in Chinese, have long been utilized as both edible and medicinal plants among ethnic minority groups in China, such as the Tibetan, Qiang, and Lisu peoples [11,12]. Typically, the young stems and leaves of both *C. tangutorum* and *C. macrophylla* are also consumed as special and nutritious vegetables among the Tibetan, Qiang, and Yi communities on the Qinghai–Tibet Plateau [11,12]. Several studies have identified flavonoids as one of the mainly bioactive compounds in both *C. tangutorum* and *C. macrophylla* [11,12,13]. However, due to the insufficient knowledge about their phytochemicals and health benefits, the industrial utilization of these species in food and pharmaceutical sectors remains limited. In fact, our preliminary analysis revealed that both *C. tangutorum* and *C. macrophylla* contain substantial amounts of pectic polysaccharides (approximately 5–6%, *w/w*). However, the structural characteristics and biological properties of these pectic polysaccharides in both *C. tangutorum* and *C. macrophylla* have never been studied and compared, which significantly hinders their potential industrial applications.

Therefore, to promote the industrial utilization of *C. tangutorum* and *C. macrophylla* and their derived pectin molecules, this study systematically characterized the structural and biological properties of pectins isolated from both *C. tangutorum* and *C. macrophylla*. The findings can provide valuable insights for developing these pectins as functional ingredients in nutraceutical and pharmaceutical applications.

## 2. Materials and Methods

### 2.1. Raw Materials and Reagents

The tender leaves of Shigecai (*C. macrophylla* Willd. and *C. tangutorum* O. E. Schulz) were harvested from the Aba Tibetan and Qiang Autonomous Prefecture and the Ganzi Tibetan Autonomous Prefecture in Sichuan Province on 16 June 2024. The specimens were authenticated by Professor Yuan Liu from Southwest Minzu University and stored in the Qinghai–Tibetan Plateau Ethnic Medicinal Resources Protection and Utilization Key Laboratory of National Ethnic Affairs Commission of the People’s Republic of China. All samples were lyophilized via vacuum freeze–drying for 48 h, and then pulverized and sieved through a 60-mesh sieve.

Enzymes utilized for removing proteins and starch during the preparation of RG-I-enriched pectin from Shigecai leaves, including pancreatin (≥4000 U/g), heat-stable α-amylase (≥40,000 U/g), and amyloglucosidase (≥100,000 U/g), were purchased from the supplier Solarbio (Beijing, China). Chemical reagents utilized for the extraction and characterization of RG-I-enriched pectin from Shigecai leaves, including choline chloride, ethylene glycol, ethanol, standardized monosaccharides, 1-phenyl-3-methyl-5-pyrazolone (PMP), potassium bromide (KBr), deuterium oxide (D_2_O), and sodium chloride (NaCl), were collected from the supplier Sigma (St. Louis, MO, USA). In addition, reagents utilized for the evaluation of biological properties, including aminoguanidine (AG), DMEM medium, MTT, 2,2′-azino-bis-(3-ethylbenzothiazoline-6-sulfonic acid (ABTS), TAK-242, TLR2-IN-C29, and commercial ELISA kits, were obtained from the suppliers Sigma, MCE China (Shanghai, China), and Elabscience (Wuhan, China). Four probiotics, including *Bifidobacterium adolescentis*, *Lactobacillus plantarum*, *Lactobacillus fermentum*, and *Lactobacillus rhamnosus*, were obtained from the China General Microbiological Culture Collection Center (Beijing, China) and the American Type Culture Collection (Manassas, VA, USA).

### 2.2. Preparation of RG-I-Enriched Pectin from Shigecai Leaves

**Figure 1:**
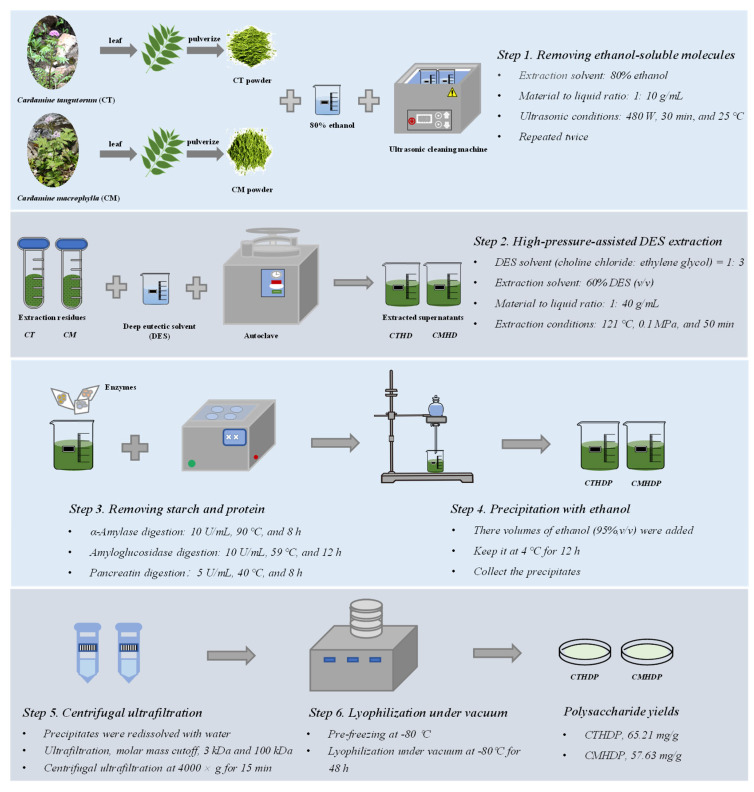
Flowchart for the extraction and purification of RG-I-enriched pectin from Shigecai leaves. CTHDP and CMHDP indicate RG-I-enriched pectins isolated from *C. tangutorum* and *C. macrophylla*, respectively.

Previous studies have revealed that high-pressure-assisted deep eutectic solvent (DES) extraction (HPDEE) can efficiently and selectively extract pectic polysaccharides with enhanced biological functions [14,15]. Therefore, RG-I-enriched pectin was extracted from Shigecai leaves using the HPDEE method, following formerly developed protocols with modifications [14,15]. The detailed procedure, outlined in Figure 1, consists of six steps: (1) ultrasonic-assisted ethanol extraction to remove ethanol-soluble components (conditions: 80% ethanol, 1: 10 g/mL, 480 W, 30 min, and 25 °C); (2) high-pressure-assisted DES extraction for RG-I-enriched pectin extraction (conditions: 60% (*v/v*) DES solution (choline chloride: ethylene glycol, 1:3 molar ratio), 1: 40 g/mL, 121 °C, 0.1 MPa, and 50 min); (3) sequential enzymatic digestion to eliminate starch and protein (α-amylase digestion, 10 U/mL, 90 °C, and 8 h; amyloglucosidase digestion, 10 U/mL, 59 °C, and 12 h; pancreatin digestion, 5 U/mL, 40 °C, and 8 h), (4) ethanol precipitation of RG-I-enriched pectin with three volumes of ethanol; (5) isolation of RG-I-enriched pectin by centrifugal ultrafiltration (molar mass cutoffs: 100 kDa and 3 kDa); (6) lyophilization of RG-I-enriched pectin under vacuum (−80 °C, 48 h). Finally, the partially purified RG-I-enriched pectin molecules of *C. tangutorum* and *C. macrophylla* were designated as CTHDP and CMHDP, respectively.

### 2.3. Chemical and Structural Characterization of RG-I-Enriched Pectin from Shigecai Leaves

The chemical and structural characteristics of CTHDP and CMHDP were characterized. Briefly, chemical composition analysis of CTHDP and CMHDP was performed using established colorimetric methods: total polysaccharide content (phenol–sulfuric acid method), total uronic acid content (*m*-hydroxyphenyl assay), total protein content (Bradford’s assay), and total bound polyphenol content (Folin–Ciocalteu’s method) [16,17,18,19]. Molecular weight distributions of CTHDP and CMHDP were determined using an established protocol involving high-performance size exclusion chromatography collected with multi-angle laser light scattering and refractive index detection (HPSEC-MALLS-RID, Wyatt Technology Co., Santa Barbara, CA, USA) [14]. Separation was performed on a Shodex OHpak SB-804 HQ column (8.0 × 300 mm) with 0.9% NaCl mobile phase in isocratic mode (0.5 mL/min and 30 °C). Samples (~1.0 mg/mL) were injected (100 μL), with data acquisition and analysis conducted using Astra software (v7.1.3). High-performance liquid chromatography (HPLC, Shimadzu, Tokyo, Japan) following acid hydrolysis and PMP derivatization was employed for quantifying monosaccharides released from CTHDP and CMHDP, following a formerly reported procedure [14]. Each sample (~6.0 mg/mL) underwent acid hydrolysis (4 M TFA, 95 °C, and 12 h), followed by PMP derivatization (0.6 M NaOH, 0.5 M PMP, 70 °C, and 100 min). PMP derivatives were separated isocratically on a ZORBAX Eclipse XDB-C18 column (4.6 × 250 mm, 5 µm) with acetonitrile–phosphate buffer (17: 83 (*v/v*), 1.0 mL/min, and 30 °C) and detected at 245 nm (20 µL injection). A mixed standard containing mannose, rhamnose, glucuronic acid, galacturonic acid, glucose, galactose, xylose, and arabinose was used to identify monosaccharide compositions in samples. Fourier transform infrared (FT-IR) spectroscopy (PerkinElmer, Waltham, MA, USA) was employed for profiling functional groups and determining degree of esterification (DE) of CTHDP and CMHDP [14]. Each sample (~1.0 mg) was mixed with KBr (100 mg), pressed into pellets, and scanned from 4000 to 400 cm^−1^. The level of DE was calculated using the signals at ~1744.8 cm^−1^ and 1623.1 cm^−1^. Finally, a Bruker Ascend 600 MHz nuclear magnetic resonance (NMR) spectrometer (Bruker, Rheinstetten, Germany) was employed for profiling glycosidic linkage patterns of CTHDP and CMHDP [14]. Each sample (~40.0 mg) was added into 1.0 mL of D_2_O overnight and subjected to NMR analysis at 600.13 MHz (^1^H) and 150.90 MHz (^13^C). More detailed procedures of each method are supplied in the Supplementary Materials (Section S1).

### 2.4. Evaluation of Biological Properties of RG-I-Enriched Pectin from Shigecai Leaves

To assess the biological activities of CTHDP and CMHDP from Shigecai leaves, in vitro antioxidant ability, antiglycation activity, prebiotic effect, and immunostimulatory effect of both samples were studied. Specifically, their ABTS free radical scavenging capacity and ferric reducing antioxidant power (FRAP) were measured to evaluate antioxidant capacity, following reported procedures [14,20]. For FRAP evaluation, 100 µL of sample solutions (2.0–10.0 mg/mL) was combined with 100 µL of 1% (*w/v*) potassium ferricyanide, heated at 50 °C (20 min), then treated with 100 µL of 10% (*w/v*) trichloroacetic acid and centrifuged. After adding 300 µL of ultrapure water and 60 µL of 0.1% (*w/v*) ferric chloride, the absorbance was detected at 700 nm. ABTS radical scavenging capacity was assessed by mixing 20 μL of sample solutions (2.0–10.0 mg/mL) with 200 μL of ABTS working solution in 96-well plates. After incubation (37 °C, 6 min), the absorbance was detected at 734 nm. IC_50_ values (mg/mL) were calculated using logarithmic regression. In addition, their inhibitory effects on advanced glycosylation end product (AGE) formation were analyzed to determine antiglycation activity, following a formerly established protocol [21]. The sample group contained sodium azide (0.5%, *w/v*), BSA (3%, *w/v*), glucose (500 mM), and sample solutions (0.25–4.0 mg/mL). After 14-day incubation at 37 °C in the dark, the fluorescence intensity was detected at λ_ex_/λ_em_ = 370/440 nm. Moreover, their growth-promoting effects on selected probiotics (*Bifidobacterium adolescentis*, *Lactobacillus plantarum*, *Lactobacillus fermentum*, and *Lactobacillus rhamnosus*) were tested to assess prebiotic potential, according to a published method [21]. Each sample (10 mg/mL) was added to carbohydrate-free MRS medium, followed by inoculation with respective strains. *L. plantarum* and *B. adolescentis* were cultivated anaerobically at 37 °C for 48 h, *L. fermentum* anaerobically at 30 °C (48 h), and *L. rhamnosus* aerobically at 37 °C (48 h). OD_600_ was then measured for all cultures. Finally, their modulatory effects on mediator/cytokine production in RAW 264.7 macrophages were examined to evaluate immunostimulatory activity, following prior procedures [21]. RAW 264.7 macrophages were seeded in 24-well plates (1 × 10^5^ cells/well) and incubated at 37 °C/5% CO_2_ for 16 h. After supernatant removal, cells were treated with samples (100–400 μg/mL, 1.0 mL) for 48 h, using culture medium (blank control) and 1 μg/mL LPS (positive control). Supernatants were reacted with Griess’s reagent (I + II, RT) and absorbance measured at 540 nm, with NaNO_2_ standards quantifying nitric oxide (NO). Cytokine levels were detected by commercial ELISA kits (Elabscience, Wuhan, China) per manufacturer’s protocol. Additionally, the influence of specific TLR4 inhibitor (TAK-242) and TLR2 inhibitor (TLR2-IN-C29) on CTHDP- and CMHDP-induced mediator/cytokines production in RAW 264.7 macrophages was investigated to clarify whether these pectin molecules enhance immune responses via TLR4/TLR2-mediated signaling pathways. More detailed procedures of each method are supplied in the Supplementary Materials (Section S2).

### 2.5. Statistical Analysis

All data are presented as mean ± deviation from three independent replicates. Statistical analyses were performed using Origin 9.0 software, with significance (*p* < 0.05) evaluated through two-tailed Student’s *t*-test or one-way analysis of variance (ANOVA).

## 3. Results and Discussion

### 3.1. Chemical and Structural Characteristics of CTHDP and CMHDP

#### 3.1.1. Primary Chemical Properties of CTHDP and CMHDP

The biological properties of pectin molecules, such as antioxidant and immunoregulatory effects, are strongly influenced by their primary chemical properties, particularly total uronic acid content and bound polyphenol content [5,22,23]. However, these parameters are still unknown in Shigecai pectin, which ultimately restricts their applications in the food industry. Hence, to facilitate their industrial utilization, the primary chemical properties of CTHDP and CMHDP were investigated. Table 1 presents the yields and primary chemical properties of CTHDP and CMHDP. Notably, CTHDP and CMHDP, confirmed as RG-I-enriched pectin through later composition monosaccharide and NMR analysis (Section 3.1.3) yielded 65.21 mg/g and 57.63 mg/g, respectively. The extraction yields of pectin molecules in Shigecai leaves (about 5.75–6.52%, *w/w*) are comparable to those of mulberry leaves (about 5.32–6.92%, *w/w*) [24,25], and are markedly higher than sweet tea leaves (about 3.65–4.64%, *w/w*) [15] and lotus leaves (about 0.97–2.65%, *w/w*) [26,27]. These results demonstrate that Shigecai leaves are potential resources for extracting RG-I-enriched pectin molecules. In addition, CTHDP and CMHDP exhibited extremely high total polysaccharide contents (90.16–92.51 mg/100 mg) and relatively low total protein contents (1.28–1.8 mg/100 mg). Furthermore, both CTHDP and CMHDP contained relatively high levels of total uronic acids, ranging from 24.91 mg/100 mg (CMHDP) to 31.68 mg/100 mg (CTHDP), suggesting that these polysaccharides contained pectic polysaccharides, similar to previous reports [14,20,27]. Notably, a higher uronic acid content is linked to enhanced biological activity due to increased binding sites and interaction potentials, probably leading to stronger immunomodulatory and antioxidant effects [5,23]. Moreover, despite the sequential extraction, precipitation, and centrifugal ultrafiltration conducted to remove polyphenols (Figure 1), trace amounts of bound polyphenols (8.21–9.82 mg GAE/g) remained in both CTHDP and CMHDP. These residual polyphenols probably enhance the antioxidant capacity of the pectin molecules [23]. Collectively, the results indicate that Shigecai leaves are a promising source for extracting RG-I-enriched pectin, with CTHDP and CMHDP exhibiting minimal variations in their primary chemical properties.

#### 3.1.2. Molecular Weights and Functional Groups of CTHDP and CMHDP

**Figure 2:**
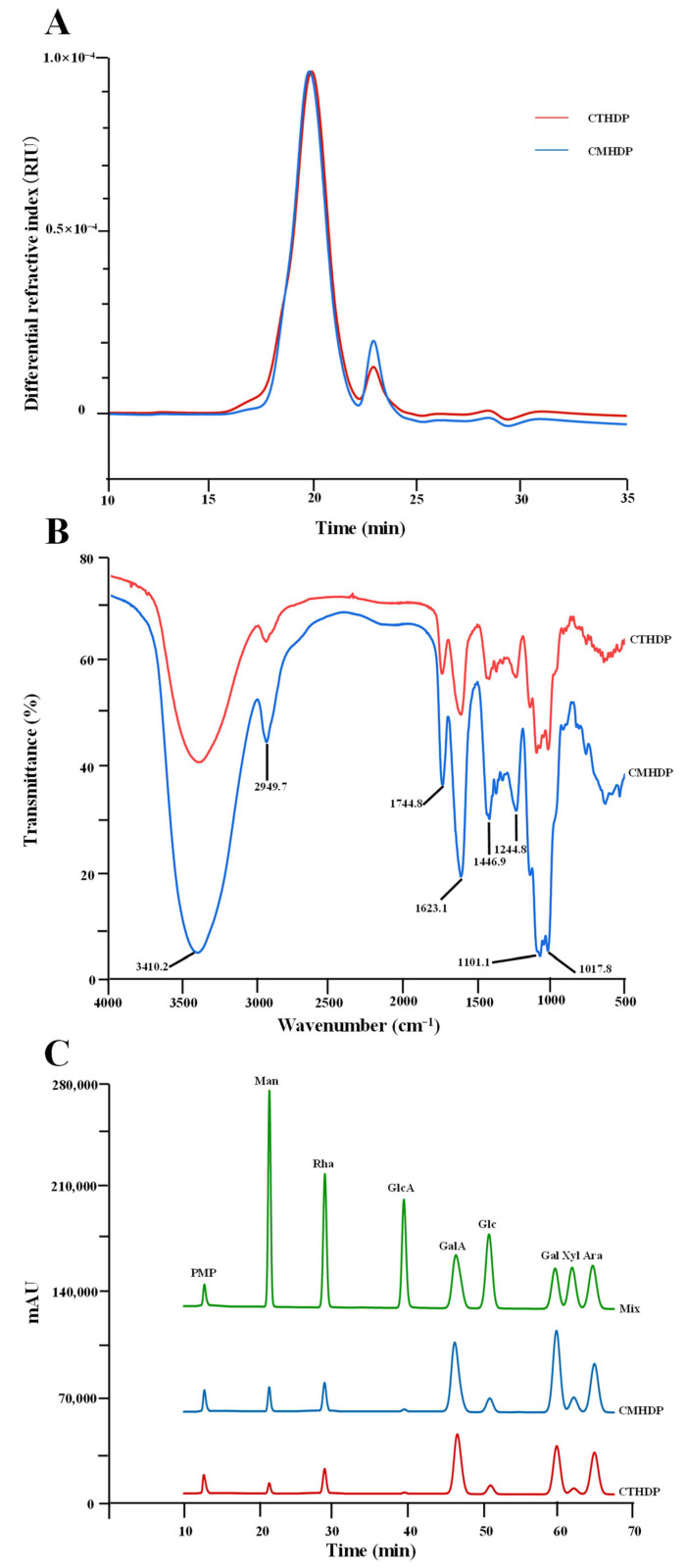
Size exclusion chromatographic elution curves (**A**), FT-IR spectra (**B**), and HPLC elution curves of constituent monosaccharides (**C**) of CTHDP and CMHDP. CTHDP and CMHDP indicate RG-I-enriched pectins isolated from *C. tangutorum* and *C. macrophylla*, respectively; the codes for individual monosaccharides are the same as in Table 1.

Apart from the primary chemical properties, the biological properties of pectin molecules, such as antioxidant ability, immunoregulatory activity, and anti-inflammatory effect, are also affected by their molecular weight distribution and degree of esterification (DE) [2,5,9]. Nevertheless, the molecular weights and functional groups of CTHDP and CMHDP have not yet been systematically characterized. To address this gap, we conducted an in-depth investigation of the molecular weights and functional group profiles of CTHDP and CMHDP in this study. Figure 2A displays the comparable HPSEC elution curves of CTHDP and CMHDP. Notably, CTHDP and CMHDP exhibited nearly identical symmetrical elution patterns, with similar elution time, suggesting comparable molecular weight distributions. Quantitatively, their molecular weights ranged from 3.33 × 10^4^ Da (CTHDP) to 4.12 × 10^4^ Da (CMHDP), with polydispersity indexes of 2.52 (CTHDP) and 2.21 (CMHDP), respectively. These values align with RG-I-enriched pectin from jujube fruits (about 2.88 × 10^4^–4.09 × 10^4^ Da) and sweet leaves (about 4.268 × 10^4^–4.472 × 10^4^ Da) [20,28], but are markedly lower than those reported for lotus leaves (about 4.476 × 10^4^–1.04 × 10^5^ Da) [26], wolfberry (about 8.638 × 10^4^–9.277 × 10^4^ Da) [29], young apple (about 5.92 × 10^5^–1.787 × 10^6^ Da) [30], and citrus peel (about 9.506 × 10^4^–2.813 × 10^5^ Da) [31]. Generally, the lower molecular weight pectins are more easily absorbed and metabolized by biological systems, thereby potentially enhancing their bioactivity [5].

Figure 2B displays the comparable FT-IR spectra of CTHDP and CMHDP, which exhibited nearly identical profiles and characteristic absorption bands, indicating similar functional group compositions. Both samples showed characteristic signals corresponding to pectin molecules, including 3410.2, 2949.7, 1744.8, 1623.1, 1446.9, 1244.8, 1101.1, and 1017.8 cm^−1^, consistent with previously reported RG-I-enriched pectin values [14,20,30,32,33,34]. In particular, the characteristic peaks at 1744.8 cm^−1^ and 1623.1 cm^−1^ can be utilized for the estimation of the relative DE value [20,28]. Based on their peak area calculations, the relative DE values were determined to be 35.91% for CTHDP and 33.01% for CMHDP, indicating both as low-methoxyl pectins. Generally, pectin with lower DE levels typically contained more free carboxyl groups, leading to improved solubility and enhanced bioactivity [21,28].

#### 3.1.3. Monosaccharide Compositions and Glycosidic Linkage Patterns of CTHDP and CMHDP

The galacturonic acid ratio, the HG/RG-I domain ratio, the side chain pattern, and the branching degree of pectin largely determine its functionality and bioactivity [2,5,7,9]. Therefore, to elucidate the chemical structures of CTHDP and CMHDP, we conducted comprehensive monosaccharide composition and glycosidic linkage pattern analyses. Figure 2C displays comparable HPLC profiles of compositional monosaccharides released from CTHDP and CMHDP, exhibiting nearly identical elution patterns that suggest similar monosaccharide compositions. Quantitatively, as summarized in Table 1, galacturonic acid (31.66–35.29 mol%), galactose (23.23–28.12 mol%), arabinose (18.48–22.40 mol%), and rhamnose (6.77–8.85 mol%) were identified as major components in both CTHDP and CMHDP, similar to previously reported RG-I-enriched pectin values [20,26,28,30,31,35]. Based on the molar percentages of these monosaccharides [26,29], it could be inferred that CTHDP and CMHDP had comparable molar percentages of RG-I (60.14–63.33 mol%) and HG (24.89–26.44 mol%) domains (Table 1). Furthermore, the degrees of RG-I branching of CTHDP and CMHDP were calculated as (Gal + Ara)/Rha, with calculated ratios of 5.16 and 6.88 for CTHDP and CMHDP, indicating highly branched side chains in both pectins.

**Figure 3:**
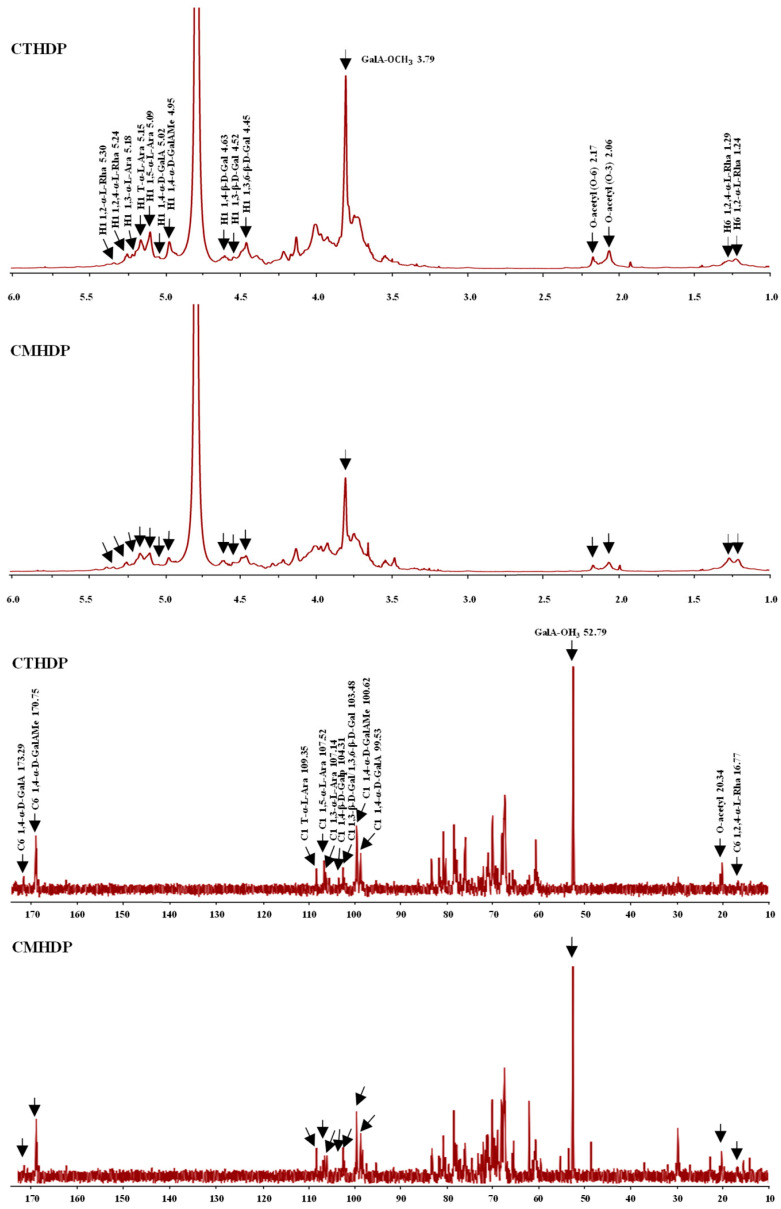
^1^H and ^13^C NMR spectra of CTHDP and CMHDP. CTHDP and CMHDP indicate RG-I-enriched pectins isolated from *C. tangutorum* and *C. macrophylla*, respectively.

Furthermore, to elucidate the glycosidic linkage patterns of CTHDP and CMHDP, ^1^H and ^13^C NMR spectroscopy analyses were conducted. As presented in Figure 3, the NMR spectra of both samples exhibited highly similar profiles, suggesting closely related glycosidic linkage patterns. Notably, characteristic signals for the main chains and branched chains of HG and RG-I pectin regions were identified in CTHDP and CMHDP, further confirming their structural similarity. For instance, the distinct chemical shifts at 5.02 (H-1)/99.53 (C-1)/173.26 ppm (C-6), 4.95 (H-1)/100.62 (C-1)/170.75 ppm (C-6), 5.30 (H-1)/1.24 (H-6), and 5.24 (H-1)/1.29 (H-6)/16.77 ppm (C-6), probably corresponded to 1,4-α-D-GalA*p*, 1,4-α-D-GalAMe*p*, 1,2-α-L-Rha*p*, and 1,2,4-α-L-Rha*p*, respectively, which represented the backbones of HG and RG-I pectin regions in both CTHDP and CMHDP [20,28,36,37,38]. In addition, the distinct chemical shifts at 4.52 (H-1)/103.48 ppm (C-1), 4.45 (H-1)/103.48 ppm (C-1), 4.63 (H-1)/104.31 ppm (C-1), 5.15 (H-1)/109.35 ppm (C-1), 5.18 (H-1)/107.14 ppm (C-1), and 5.09 (H-1)/107.52 ppm (C-1), probably corresponded to 1,3-β-D-Gal*p*, 1,3,6-β-D-Gal*p*, 1,4-β-D-Gal*p*, T-α-L-Ara*f*, 1,3-α-L-Ara*f*, and 1,5-α-L-Ara*f*, respectively, which represented the sidechains of RG-I pectin region in both samples [20,28,36,37,38]. Furthermore, the distinct chemical shifts at 3.79/52.79 ppm and 2.06/2.17/20.34 ppm indicated the methyl- and acetyl-esterified groups in both CTHDP and CMHDP [20,38]. Taken together, these results infer that CTHDP and CMHDP possess comparable glycosidic linkage patterns, mainly composed of HG and RG-I pectin domains with arabinogalactan and arabinan branched chains.

### 3.2. Antioxidant and Antiglycation Effects of CTHDP and CMHDP

**Figure 4:**
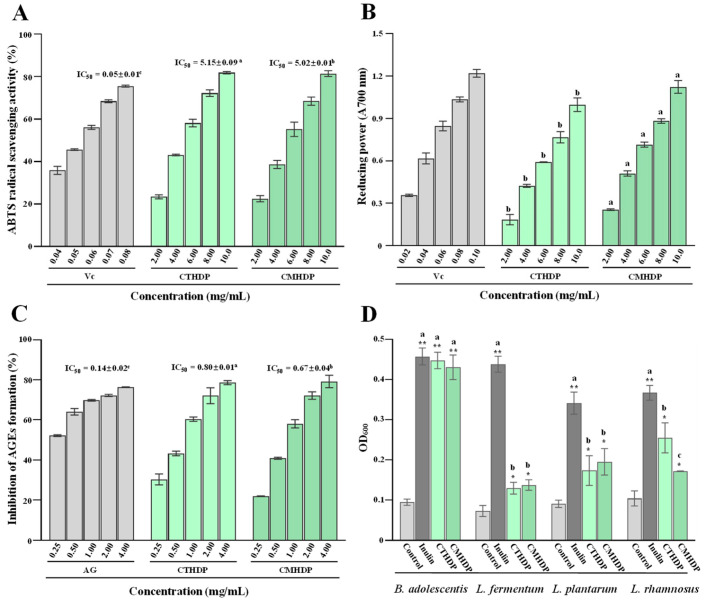
In vitro antioxidant (**A**,**B**), antiglycation (**C**), and prebiotic effects (**D**) of CTHDP and CMHDP. CTHDP and CMHDP indicate RG-I-enriched pectins isolated from *C. tangutorum* and *C. macrophylla*, respectively; the error bars are standard deviations; significant differences (*p* < 0.05) between CTHDP and CMHDP are shown by data bearing different letters; significant differences in the growth rates of individual probiotics between samples and negative control are shown by * *p* < 0.05, ** *p* < 0.01.

Pectin demonstrates remarkable antioxidant activity by effectively scavenging free radicals, chelating metal ions, and inhibiting reactive oxygen species production, which is closely intertwined with its structural properties [5,6]. Therefore, to advance the application potential of Shigecai and its derived RG-I-enriched pectin, the antioxidant and antiglycation effects of CTHDP and CMHDP were systematically characterized. As displayed in Figure 4A,B, both CTHDP and CMHDP exhibited dose-dependent ABTS radical scavenging ability and reducing power (FRAP value). Notably, CMHDP demonstrated superior antioxidant activity over CTHDP. The IC_50_ values of the ABTS scavenging ability for CTHDP and CMHDP were 5.15 mg/mL and 5.02 mg/mL, respectively. CMHDP exhibited significantly higher reducing power across all tested concentrations than that of CTHDP, particularly at 10.0 mg/mL (absorbance at 700 nm, 1.12 for CMHDP and 1.01 for CTHDP). Additionally, Shigecai-derived RG-I-enriched pectin exhibits antioxidant potency comparable to reported sources: hawthorn pectin (reducing power at 10 mg/mL ranged from 0.315 to 0.349) [39], citrus peel pectin (ABTS IC_50_ ranged from 2.06 to 9.11 mg/mL) [40], and black mulberry pectin (ABTS scavenging rate at 4.0 mg/mL ranged from 27.82% to 56.58%) [41]. This demonstrates its potential as a natural antioxidant for industrial food applications.

Furthermore, pectin scavenges radicals or inhibits their formation via metal chelation. Pectin’s antioxidant potency is strongly linked to its bound polyphenol content, uronic acid content, DE value, and molecular weight, with bound polyphenols being the most critical factor [23]. These bound polyphenols significantly contribute to pectin’s antioxidant activity, enabling the scavenging of DPPH and ABTS radicals [23]. Recent studies have confirmed that pectin’s antioxidant activity positively correlates with bound polyphenol content [14,23], but negatively correlates with DE value [21,28]. Thus, taken together, the superior antioxidant activity of CMHDP compared with CTHDP is likely primarily attributed to the combined effects of its bound polyphenol content, DE value, and uronic acid content.

To assess the antiglycation potency of CTHDP and CMHDP, we evaluated their inhibitor effects on advanced glycation end product (AGE) formation. Figure 4C compares their inhibitory effects on AGE formation. Both samples exerted marked antiglycation activity, with CMHDP (IC_50_ value, 0.67 mg/mL) outperforming CTHDP (IC_50_ value, 0.80 mg/mL). Notably, the antiglycation activity trend of both samples paralleled their antioxidant activity, suggesting a strong correlation between these two activities. In fact, pectin can scavenge free radicals and active dicarbonyl components during AGE formation owing to its free radical scavenging capacity [42]. Thus, this suggests that the antiglycation effect of Shigecai-derived RG-I-enriched pectin is closely linked to its antioxidant activity.

### 3.3. Prebiotic Effects of CTHDP and CMHDP

Pectin resists digestion in the upper gastrointestinal tract (mouth, stomach, and small intestine), but is metabolized and utilized by gut microbiota in the colon, thereby regulating intestinal microbial composition and microbial metabolites to demonstrate prebiotic activity [2,3,5,6]. Particularly, *Lactobacillus* and *Bifidobacterium* spp. are best understood for their applications as probiotics [43]. Therefore, to evaluate the prebiotic potential of CTHDP and CMHDP, we assessed their growth-promoting effects on four individual probiotic strains in vitro, including *Lactobacillus fermentum*, *Lactobacillus plantarum*, *Lactobacillus rhamnosus*, and *Bifidobacterium adolescentis*.

As presented in Figure 4D, both samples demonstrated marked prebiotic potential by enhancing the growth rates of different probiotic strains (*Lactobacillus fermentum*, *Lactobacillus plantarum*, *Lactobacillus rhamnosus*, and *Bifidobacterium adolescentis*), with particularly pronounced effects on *B. adolescentis*. The promotive effects of both samples on *B. adolescentis* growth were comparable to that of inulin (a representative commercial prebiotic), indicating that RG-I-enriched pectin derived from Shigecai exhibits excellent potential for the development as a targeted prebiotic for *B. adolescentis*. Notably, *Bifidobacterium* spp. strains are established as safe and effective probiotics [44], and *B. adolescentis* represents one of the most abundant species within this genus in the healthy human intestinal microbiota. Collectively, these results suggest that Shigecai-derived RG-I-enriched pectin holds good potential for development as a prebiotic targeting *Bifidobacterium* spp. Nevertheless, animal studies are required to confirm their prebiotic effect on intestinal microbial composition in future, given the limitations of in vitro systems.

### 3.4. Immunostimulatory Effects of CTHDP and CMHDP

**Figure 5:**
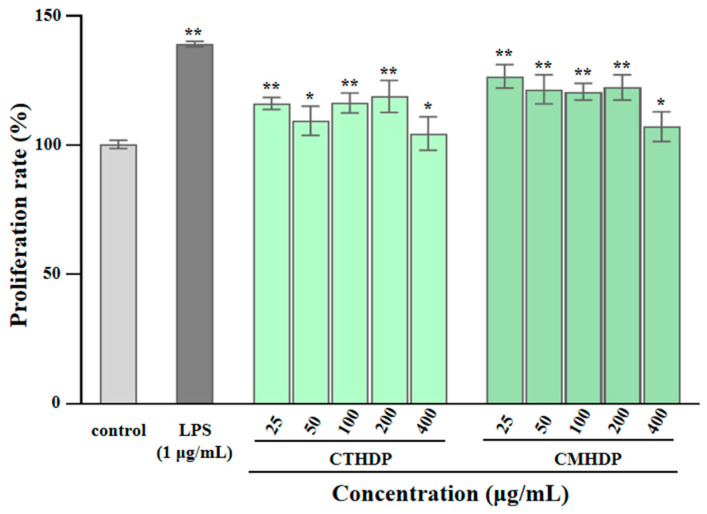
Effects of CTHDP and CMHDP on the proliferation of RAW 264.7 macrophages. CTHDP and CMHDP indicate RG-I-enriched pectins isolated from *C. tangutorum* and *C. macrophylla*, respectively; the error bars are standard deviations; significant differences between sample groups and control group are shown by * *p* < 0.05 and ** *p* < 0.01.

**Figure 6:**
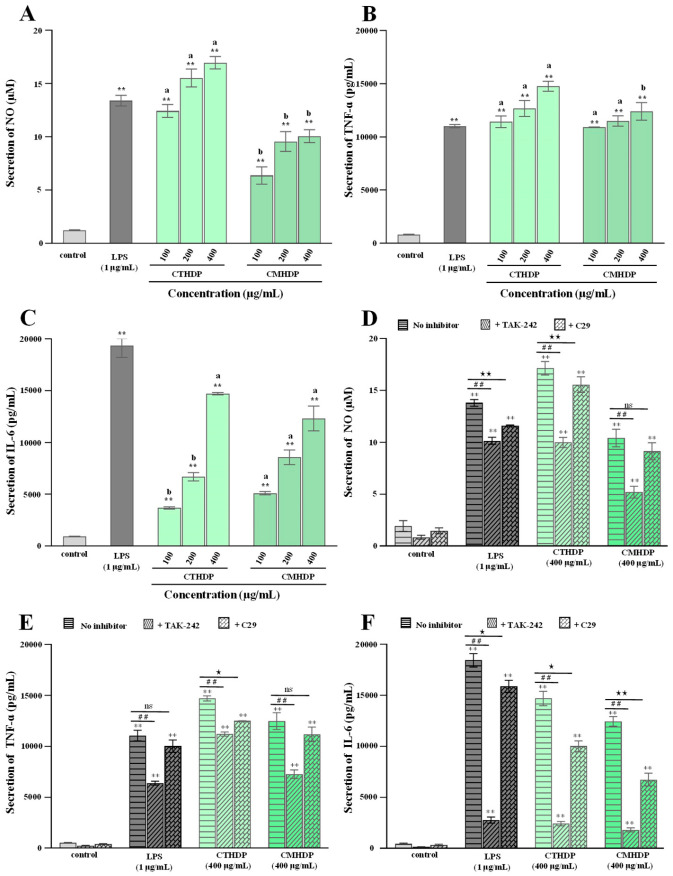
Immunoregulatory effects (**A**–**C**) of CTHDP and CMHDP and effects of selective inhibitors (**D**–**F**) on their immune responses induced by CTHDP and CMHDP. (**A**–**C**) indicate the nitric oxide (NO) production, tumor necrosis factor-α (TNF-α) secretion, and interleukin-6 (IL-6) secretion in macrophages induced by CTHDP and CMHDP; D, E, and F indicate the impacts of selective inhibitors (TAK-242 and C29) on the production of NO, TNF-α, and IL-6 in macrophages induced by CTHDP and CMHDP. CTHDP and CMHDP indicate RG-I-enriched pectins isolated from *C. tangutorum* and *C. macrophylla*, respectively; the error bars are standard deviations; significant differences (*p* < 0.05) between CTHDP and CMHDP are shown by data bearing different letters (a–b); significant differences between sample groups and negative control group are shown by ** *p* < 0.01. Significant differences between samples treated with TAK-242 and without TAK-242 are shown by ^##^ *p* < 0.01. Significant differences between samples treated with C29 and without C29 are shown by ^★^ *p* < 0.05 and ^★★^ *p* < 0.01. ‘ns’ indicates no significant difference.

Pectin molecules, particularly RG-I-enriched pectin derived from vegetables and fruits, exhibit notable immunoregulatory activity towards macrophages, lymphocytes, and the complement system [5,6,10]. Macrophages, particularly RAW 264.7 cells, have been widely applied to evaluating in vitro immunoregulatory effects [22]. Therefore, to evaluate their potential applications as natural immune modulators, we assessed the immunostimulatory effects of RG-I-enriched pectin derived from Shigecai leaves on RAW 264.7 macrophages. As presented in Figure 5, neither CTHDP nor CMHDP exhibited toxicity towards RAW 264.7 macrophages at any tested concentration. Figure 6A–C demonstrate the regulator effects of both samples on NO production (Figure 6A), TNF-α secretion (Figure 6B), and IL-6 secretion (Figure 6C) in macrophages. Notably, both samples significantly enhanced the production of NO, TNF-α, and IL-6, indicating a potent immunoenhancing effect. In particular, at a concentration of 400 μg/mL, CTHDP exhibited superior immunoenhancing effects compared with CMHDP, with higher levels of NO (16.97 μM vs. 10.07 μM), TNF-α (14,768.6 pg/mL vs. 12,411.4 pg/mL), and IL-6 (14,710 pg/mL vs. 12,322.5 pg/mL). Generally, the immunoregulatory effects of pectins strongly associate with their chemical and structural properties. These effects exhibit negative correlations toward molecular weight and DE level [28,45,46], but positive correlations toward uronic acid content and RG-I domain abundance [10,14,47]. Thus, taken together, the superior immunoregulatory effect of CTHDP might be partially attributed to the synergistic contributions of its higher uronic acid content, lower molecular weight, and greater RG-I domain ratio. Nevertheless, due to its indigestibility in the upper GI tract, animal studies are needed to confirm whether CTHDP and CMHDP exert immunostimulatory effects via gut microbiota modulation in future.

Pectin activates macrophages and enhances immune responses through recognition using pattern recognition receptors, such as toll-like receptors (TLRs) [5,46]. So, to further elucidate the possible mechanism of CTHDP- and CMHDP-mediated macrophage activation, we assessed the effects of selective TLR4 and TLR2 inhibitors (TAK-242 and C29, respectively) on their immunoregulatory activity. Figure 6D–F display the effects of TAK-242 and C29 on NO, TNF-α, and IL-6 release in macrophages. Notably, both TAK-242 (1 μM) and C29 (1 μM) inhibitors markedly inhibited the immune responses induced by CTHDP and CMHDP (Figure 6D–F). In particular, TAK-242 exhibited a stronger inhibitory effect than C29, indicating that the immunoregulatory effects of both samples depend more crucially on TLR4 than TLR2, similar to reported results [46,48].

## 4. Conclusions

The present findings suggest that Shigecai leaves are promising sources of RG-I-enriched pectin. Both CTHDP and CMHDP exhibited highly similar chemical and structural properties, dominated by the RG-I pectin region (60.14–63.33 mol%). Furthermore, both CTHDP and CMHDP demonstrated remarkable antioxidant, antiglycation, prebiotic, and immunoregulatory effects, closely linked to their bound polyphenol content, uronic acid level, and molecular weight. These findings provide good evidence for the development of RG-I-enriched pectin from Shigecai as a functional food ingredient. However, further studies using animal models are needed to validate these beneficial effects, due to the inherent limitations of in vitro assays. Additionally, fine structural characterization and modification are required to elucidate the precise structure–function relationships of these RG-I-enriched pectic polysaccharides.

## Figures and Tables

**Table 1:** Extraction yields and physicochemical characteristics of RG-I-enriched pectin from Shigecai leaves.

	CTHDP	CMHDP
Extraction yield (mg/g)	65.21 ± 0.41 ^a^	57.63 ± 0.13 ^b^
Total polysaccharide content (mg/100 mg)	92.51 ± 0.63 ^a^	90.16 ± 1.57 ^a^
Total uronic acid content (mg/100 mg)	31.68 ± 2.27 ^a^	24.91 ± 2.77 ^b^
Total protein content (mg/100 mg)	1.28 ± 0.09 ^b^	1.80 ± 0.14 ^a^
Bound polyphenol content (mg GAE/g)	8.21 ± 0.21 ^b^	9.82 ± 0.21 ^a^
Degree of esterification (%)	35.91 ± 1.32 ^a^	33.01 ± 2.11 ^a^
*M_w_* × 10^4^ (Da, error)	3.33 ± 0.01 ^b^	4.12 ± 0.02 ^a^
*M_w_/M_n_*	2.52	2.21
Monosaccharides and molar percentages (mol%)		
Galacturonic acid (GalA)	35.29	31.66
Galactose (Gal)	23.23	28.12
Arabinose (Ara)	22.40	18.48
Rhamnose (Rha)	8.85	6.77
Glucose (Glc)	4.47	5.26
Xylose (Xyl)	3.29	5.95
Mannose (Man)	1.96	3.20
Glucuronic acid (GlcA)	0.51	0.56
HG (mol%)	26.44	24.89
RG-I (mol%)	63.33	60.14
(Gal + Ara)/Rha	5.16	6.88

CTHDP and CMHDP indicate RG-I-enriched pectins isolated from *C. tangutorum* and *C. macrophylla*, respectively; data were presented as mean ± standard deviation; different letters (a–b) indicate statistical significances (*p* < 0.05) between CTHDP and CMHDP; molar percentages of HG and RG-I domains were calculated as follows: HG (mol%) = GalA (mol%) − Rha (mol%); RG-I (mol%) = 2 Rha (mol%) + Gal (mol%) + Ara (mol%).

## Data Availability

The original contributions presented in the study are included in the article/Supplementary Material, further inquiries can be directed to the corresponding authors.

## Supplementary Materials

The following supporting information can be downloaded at: https://www.mdpi.com/article/10.3390/foods14132340/s1, Section S1: Chemical and structural characteristics of RG-I-enriched pectin from Shigecai leaves (CTHDP and CMHDP); Section S2: Evaluation of antioxidant, antiglycation, prebiotic, and immunoregulatory effects of RG-I-enriched pectin from Shigecai leaves (CTHDP and CMHDP).
