# Supplementary material for: Structural and Biological Properties of Rhamnogalacturonan-I-Enriched Pectin Isolated from Cardamine tangutorum and Cardamine macrophylla
Source: Foods. 2025 Jul 1;14(13):2340. doi: 10.3390/foods14132340 (PMC12249001; doi:10.3390/foods14132340)
Supplement: Supplementary file 1 [file foods-14-02340-s001.zip › foods-3711108-supplementary.pdf]

Supplementary materials

Structural and biological properties of rhamnogalacturonan-I-enriched pectin isolated from *Cardamine tangutorum* and *Cardamine macrophylla*

Mei-Mei Qu Mo [1, 2], Bo Li [2], Ding-Tao Wu [2, 3, *], Jing Feng [2], Jing Wei [1], Yan Wan [2], Juan Li [1], Yuan Liu [1], Wen-Bing Li [1, *]

[1] Qinghai-Tibetan Plateau Ethnic Medicinal Resources Protection and Utilization Key Laboratory of National Ethnic Affairs Commission of the People's Republic of China, Sichuan Provincial Qiang-Yi Medicinal Re-sources Protection and Utilization Technology Engineering Laboratory, Southwest Minzu University, Chengdu 610225, Sichuan, China

[2] Key Laboratory of Coarse Cereal Processing of Ministry of Agriculture and Rural Affairs, Sichuan Engi-neering & Technology Research Center of Coarse Cereal Industralization, School of Food and Biological Engineering, Chengdu University, Chengdu 610106, Sichuan, China

[3] Institute for Advanced Study, Chengdu University, Chengdu 610106, Sichuan, China

[*] Correspondence: wudingtao@cdu.edu.cn (D.T.W.); 80300039@swun.edu.cn (W.B.L.)

Section S1. Chemical and structural characteristics of RG-I-enriched pectin from Shigecai leaves (CTHDP and CMHDP)

S1.1. Chemical composition analysis of RG-I-enriched pectin from Shigecai leaves (CTHDP and CMHDP)

The total polysaccharides (mg/100 mg) in CTHDP and CMHDP were determined using the phenol–sulfuric acid method with a mixed standard of galacturonic acid (30%) and galactose (70%). In detail, 250 μL of concentrated sulfuric acid was added into 100 μL of each sample solution and shaken well, followed by the addition of 50 μL of 6% (*w/v*) phenol solution. The mixture was then incubated at 90 °C in a water bath for 10 minutes, and the absorbance of the mixture was measured at 490 nm.

The total uronic acids (mg/100 mg) in CTHDP and CMHDP were measured using the *m*-hydroxyphenyl method with galacturonic acid as a standard. In detail, 1.0 mL of sodium tetraborate–sulfuric acid solution was added into 100 μL of each sample solution, and the mixture was shaken well and incubated at 95 °C for 10 min, avoiding light. Then, 20 μL of 3-phenylphenol was added into the mixture, and the absorbance of the mixture was measured at 520 nm.

The total proteins (mg/100 mg) in CTHDP and CMHDP were detected using the Bradford method using bovine serum protein as a standard. In detail, 250 μL of Coomassie Brilliant Blue solution was added into 50 μL of each sample solution, and the mixture was incubated in the dark for 15 min, and then the absorbance of the mixture was measured at 595 nm.

The total bound polyphenols (mg GAE/g) in CTHDP and CMHDP were determined using the Folin–Ciocalteu method with gallic acid as a standard. In detail, 50 μL of each sample solution was mixed with 250 μL of Folin–Ciocalteu's reagent (0.2 mM), and incubated at room temperature for 10 min. Then the reaction was neutralized with 250 μL of Na_2CO_3 solution (20%, *w/v*), and incubated at room temperature for 30 min. Finally, the absorbance of the mixture was measured at 760 nm.

S1.2. Molecular weight distribution analysis of RG-I-enriched pectin from Shigecai leaves (CTHDP and CMHDP)

The molecular weights (M_w) and molecular weight distributions (M_w/M_n) of CTHDP and CMHDP were determined by high-performance size exclusion chromatography coupled with a multi-angle laser light scattering detector and a refractive index detector (HPSEC-MALS-RID, Wyatt Technology Co, Santa Barbara, CA, USA). In detail, HPSEC-MALS-RID measurements were carried out on an Agilent 1260 series LC system (Agilent Technologies, Palo Alto, CA, USA) with a multi-angle laser light scattering detector (MALLS, DAWN HELEOS, Wyatt Technology Co., Santa Barbara, CA, USA). An Optilab rEX refractometer (RID, DAWN EOS, Wyatt Technology Co., Santa Barbara, CA, USA) was simultaneously connected. The Shodex OHpak SB-804 HQ column (8.0 × 300 mm) was utilized for the separation of CTHDP and CMHDP, and the column temperature was set as 30 ℃. The mobile phase was 0.9% of NaCl aqueous solution at a flow rate of 0.5 mL/min. The sample concentration was about 1.0 mg/mL. An injection volume of 100 μL was

used. The Astra software (version 7.1.3, Wyatt Technology Co, Santa Barbara, CA, USA) was utilized for data acquisition and analysis.

S1.3. Constituent monosaccharide analysis of RG-I-enriched pectin from Shigecai leaves (CTHDP and CMHDP)

Constituent monosaccharides of CTHDP and CMHDP were analyzed by high-performance liquid chromatography (HPLC, L-20A, Shimadzu, Japan). In detail, 6.0 mg/mL of each sample was mixed with trifluoroacetic acid (4 M) and incubated at 95 °C for 12 h. The complete hydrolysates were evaporated until dry using rotary evaporation, and then washed with methanol three times. Afterward, 1.0 mL of ultrapure water was added to the dried hydrolysates. The hydrolysates were then mixed with sodium hydroxide (0.6 M) and 1-phenyl-3-methyl-5-pyrazolone (PMP, 0.5 M), and then the mixture was incubated at 70 °C for 100 min. Finally, the mixture was vortexed with 1 mL of chloroform. After vigorous shaking and layering, the organic phase was discarded. A mixture of glucose, galactose, galacturonic acid, glucuronic acid, rhamnose, arabinose, xylose, and mannose was prepared as a mixed standard. The mixture of acetonitrile and phosphate buffer (17: 83, *v/v*) was used as the mobile phase, and a ZORBAX Eclipse XDB-C18 column (4.6 × 250 mm, i.d. 5 μm, Agilent Technologies Inc., CA, USA) was utilized for the separation of PMP derivatives. An aliquot of 20 μL of PMP derivatives was injected into the HPLC system at the operation temperature of 30 °C, and detected at 245 nm.

S1.4. Fourier transform infrared (FT-IR) spectroscopy analysis of RG-I-enriched pectin from Shigecai leaves (CTHDP and CMHDP)

FT-IR spectra of CTHDP and CMHDP were analyzed using a Fourier infrared spectrometer (Spectrum Two, PerkinElmer, Waltham, MA, USA). Each sample (about 1.0 mg) was ground with dried KBr (100 mg), pressed, and scanned over a range of 4000 – 400 cm^{-1}. The levels of the degree of esterification (DE) of CTHDP and CMHDP were calculated according to the following equation:

$$\mathrm{DE}\ (\%) = 124.7R + 2.2013$$

$$\mathrm{R} = A1744.8/(A1744.8 + A1623.1)$$

S1.5. Nuclear magnetic resonance (NMR) spectroscopy analysis of RG-I-enriched pectin from Shigecai leaves (CTHDP and CMHDP)

One-dimensional NMR spectroscopy analysis of CTHDP and CMHDP was conducted using a Bruker Ascend 600 MHz NMR spectrometer with a z-gradient probe (Bruker, Rheinstetten, Germany). A total of 40.0 mg of each sample was accurately weighed and dissolved in 1.0 mL of D_2O overnight, and then subjected to NMR analysis. The frequencies for a proton and carbon were 600.13 and 150.90 MHz, respectively.

Section S2. Evaluation of antioxidant, antiglycation, prebiotic, and immunoregulatory effects of RG-I-enriched pectin from Shigecai leaves (CTHDP and CMHDP)

S2.1. Determination of antioxidant effects of RG-I-enriched pectin from Shigecai leaves (CTHDP and CMHDP)

The antioxidant effects of CTHDP and CMHDP, including ferric reducing antioxidant power (FRAP) and ABTS free radical scavenging capacity, were

systematically evaluated. For the evaluation of FRAP, 100 μL of each sample solution at different concentrations (2.0, 4.0, 6.0, 8.0, and 10.0 mg/mL) was mixed with 100 μL of potassium ferricyanide (1%, *w/v*), and heated in a water bath at 50 °C for 20 minutes. Then, 100 μL of trichloroacetic acid (10%, *w/v*) was added and centrifuged. Finally, 300 μL of ultrapure water and 60 μL of ferric chloride (0.1%, *w/v*) were added and the absorbance of the mixture was measured at 700 nm. For the evaluation of ABTS free radical scavenging capacity, 20 μL of each sample solution at different concentrations (2.0, 4.0, 6.0, 8.0, and 10.0 mg/mL) was mixed with 200 μL of ABTS working solution (diluted from 7 mM ABTS storage solution, and the absorbance was at 0.8 ± 0.02), and then placed into a 96-well microtiter plate. The mixed solution was heated at 37 °C for 6 min, and then the absorbance of the mixture was determined at 734 nm. Afterwards, a log-regression curve was set up for calculating IC_{50} values (mg/mL). Vitamin C (VC) was used as a positive control.

S2.2. Determination of antiglycation effects of RG-I-enriched pectin from Shigecai leaves (CTHDP and CMHDP)

To evaluate the antiglycation effects of CTHDP and CMHDP, their inhibitory effects against the formation of advanced glycosylation end products (AGEs) induced by the Maillard reaction in a BSA/Glc model were determined. The blank control group was composed of sodium azide (0.5%, *w/v*), BSA (3%, *w/v*), and glucose (500 mM). The sample group was composed of sodium azide (0.5%, *w/v*), BSA (3%, *w/v*), glucose (500 mM), and each sample solution at different concentrations of 0.25, 0.5, 1.0, 2.0, and 4.0 mg/mL. The positive control group was composed of sodium azide

(0.5%, *w/v*), BSA (3%, *w/v*), glucose (500 mM), and aminoguanidine (AG) solution at different concentrations of 0.25, 0.5, 1.0, 2.0, and 4.0 mg/mL. The mixtures were incubated at 37 °C for 14 days in the dark. Afterward, the fluorescence intensity of each group was measured under the conditions of the excitation wavelength of 370 nm and the emission wavelength of 440 nm. Afterwards, a log-regression curve was set up for calculating IC_{50} values (mg/mL).

S2.3. Determination of prebiotic effects of RG-I-enriched pectin from Shigecai leaves (CTHDP and CMHDP)

To assess the prebiotic effects of CTHDP and CMHDP on various probiotic strains, their effects on the growth of *L. fermentum* (CGMCC 1.15608), *L. plantarum* (CGMCC 1.12974), *L. rhamnosus* (ATCC 53103), and *B. adolensentis* (ATCC 15703) were determined. Each sample (10 mg/mL) was added into the carbohydrate-free MRS medium, and then each strain was inoculated into the culture medium. Both *L. plantarum* and *B. adolensentis* were cultivated at 37 °C under anaerobic conditions for 48 h, and *L. fermentum* was cultivated at 30 °C under anaerobic conditions for 48 h. *L. rhamnosus* was incubated at 37 °C for 48 h under aerobic conditions. Finally, the optical density of each group was determined at 600 nm. The carbohydrate-free MRS medium was used as a blank control and inulin was used as a positive control.

S2.4. Determination of immunoregulatory effects of RG-I-enriched pectin from Shigecai leaves (CTHDP and CMHDP)

S2.4.1. Effects of CTHDP and CMHDP on the production of nitric oxide (NO), tumor necrosis factor-α (TNF-α), and interleukin 6 (IL-6) in RAW 264.7

macrophages

An in vitro RAW 264.7 macrophage model was used to evaluate the immunoregulatory effects of CTHDP and CMHDP. In detail, to evaluate the effects of CTHDP and CMHDP on the cell viability of RAW 264.7 macrophages, RAW 264.7 macrophages were added into 96-well microtiter plates at a concentration of 5×10^4 cells/well, and incubated at 37 °C with 5% carbon dioxide for 16 h. Afterward, the supernatant of each well was removed, and 100 μL of each sample at different concentrations (25, 50, 100, 200, and 400 μg/mL) was added into the wells, and incubated for 24 h. The culture medium was used as a blank control, and 1 μg/mL of LPS solution was used as a positive control. The supernatant of each well was removed and 100 μL of MTT solution (1 mg/mL) was added. After 4 h of incubation, the supernatant of each well was removed, and 100 μL of dimethyl sulfoxide was added, and the absorbance of the mixture was measured at 570 nm. In addition, for the evaluation of the effects of CTHDP and CMHDP on the release of nitric oxide (NO), interleukin-6 (IL-6), and tumor necrosis factor-alpha (TNF-α) in RAW 264.7 macrophages, RAW 264.7 macrophages were added into a 24-well microtiter plate at a concentration of 1×10^5 cells/well, and incubated at 37 °C with 5% carbon dioxide for 16 h. The supernatant was pipetted from the wells and then incubated with 1.0 mL of each sample at different concentrations (100, 200, and 400 μg/mL) for 48 h. The culture medium was used as a blank control and 1 μg/mL of LPS solution was used as a positive control. The supernatants were then mixed with Griess I and Griess II reagents at room temperature and the absorbance of the mixture was measured at 540

nm. $NaNO_2$ was applied as a standard for calculating NO's concentration. In addition, the levels of cytokines TNF-α and IL-6 in the supernatant were measured using ELISA kits based on the manufacturer's procedure (Elabscience, Wuhan, China).

S2.4.2. Effect of TAK-242 or C29 on the production of NO, TNF-α, and IL-6 in RAW 264.7 cells induced by CTHDP and CMHDP

RAW 264.7 macrophages were added into 24-well microtiter plates at a concentration of 1 × 10^5 cells/well and incubated at 37 °C with 5% CO_2 for 16 h. After the removal of the medium, the new medium with or without TAK-242 (1 μM)/C29 (1 μM) was added into the wells and incubated for 4 h. The supernatant in the wells was aspirated out and then incubated with 1.0 mL of 400 μg/mL of CTHDP and CMHDP for 48 h. The culture medium was used as a blank control and 1 μg/mL of LPS solution was used as a positive control. Afterward, the supernatant was then mixed with Griess I and Griess II reagents at room temperature and measured at 540 nm. The $NaNO_2$ was applied as a standard for the calculation of the concentration of NO. The levels of TNF-α and IL-6 in the supernatant were measured using ELISA kits based on the manufacturer's procedures (Elabscience, Wuhan, China).
